# A model for developing the competencies of Village Health Volunteers in the prevention and control of communicable diseases at the community level: a case study of Chaiyaphum Province, Thailand

**DOI:** 10.3389/fpubh.2026.1781727

**Published:** 2026-06-25

**Authors:** Pariyakorn Chaleephrom, Songkhamchai Leethongdissakul, Vorapoj Promasatayaprot

**Affiliations:** 1 Faculty of Public Health, Mahasarakham University, Maha Sarakham, Thailand; 2 Chatturat District Public Health Office, Chaiyaphum, Thailand; 3 Faculty of Public Health, Mahasarakham University, Maha Sarakham, Thailand

**Keywords:** communicable disease control, communicable disease prevention, community, competency, Village Health Volunteers (VHVs)

## Abstract

**Introduction:**

Village Health Volunteers (VHVs) constitute a crucial community health workforce in communicable disease prevention and control. Despite generally strong performance, persistent gaps in digital literacy, data management, and risk communication indicate a need for a structured competency development model.

**Methods:**

A mixed-methods Multiphase Research design was employed from March 2022 to July 2024, integrating quantitative surveys with qualitative focus group discussions and in-depth interviews. Phase 1 comprised quantitative (*n* = 416) and qualitative (*n* = 100) data collection using multi-stage stratified random sampling and purposive sampling, respectively. Phase 2 (*n* = 34) employed the Plan–Act–Observe–Reflect cycle to develop the competency model. Phase 3 (*n* = 33) evaluated the intervention.

**Results:**

Baseline assessment indicated that most VHVs were female (80.53%), aged 51–60 years (43.03%), and had 11–20 years of experience (45.19%). Overall self-reported competency was at a high level (mean = 3.71, SD = 0.46), with strong performance in practices (mean = 4.34, SD = 0.42), moderate-to-high skills (mean = 3.60, SD = 0.45), and the lowest scores in knowledge (mean = 3.59, SD = 0.49). Qualitative findings identified substantive competency gaps in epidemiological reasoning, digital literacy, systematic data recording, risk communication, and leadership. The SMART VHV Plus Model, comprising five components (communicable disease control, management, technology, leadership and teamwork, and community health planning), was subsequently developed and delivered through five structured training programmes. Post-intervention assessment demonstrated a statistically significant improvement in overall competency scores: from a pre-intervention mean of 86.14% (SD = 7.65, classified as moderate) to a post-intervention mean of 98.16% (SD = 1.95, classified as high), representing a mean difference of 12.02 percentage points (95% CI: 9.84–14.20, *p* < 0.05).

**Discussion:**

The SMART VHV Plus Model was associated with meaningful improvements in VHV competencies in communicable disease prevention and control. Its participatory design and integration of digital literacy, leadership, and community health planning provide a potentially sustainable framework for strengthening community health workforce capacity.

## 1 Introduction

Village Health Volunteers (VHVs) are crucial to communicable disease prevention and control. They connect government public health agencies with local communities as grassroots health workers (1). In addition, they must monitor disease, screen at-risk groups, provide basic health education, follow up with patients and at-risk individuals, and provide community health statistics (2). VHVs collaborate with local public health agencies to improve disease control and containment during outbreaks like COVID-19 (3, 4). Their contributions also support universal health coverage and Thailand’s national health policies, which promote health and improve health care efficiency (5). These roles collectively position VHVs as essential actors in Thailand’s community-based communicable disease prevention and control infrastructure.

Village Health Volunteers (VHVs) face persistent challenges in several competency domains. While most VHVs demonstrate moderate to high proficiency in disease prevention and control, deficiencies in risk communication and information technology competencies remain prevalent (6). Similarly, VHVs have been found to be effective at disease surveillance and at-risk group screening but show limitations in data processing for situational analysis (7). More recently, data management and technology utilisation have been identified as the lowest-performing competency domains among VHVs (8). These converging findings underscore the need for structured competency development that addresses digital literacy and risk communication as frontline priorities. Solving these difficulties requires a systematic competency development plan that focuses on knowledge, practical skills, situation assessment, risk communication, and health network collaboration. Participatory learning approaches, engagement with public health agencies, and community support have been shown to increase VHV capacity (4, 8). Competency-based training has consistently demonstrated effectiveness in enhancing VHV knowledge, skills, and capacities for communicable disease prevention and control (9, 10). Supportive supervision, health system integration, structured training, and continuous monitoring and evaluation are established drivers of community health workforce capacity development, enabling systematic improvement in knowledge, skills, and attitudes (KSAs) (11–14).

All VHVs in Thailand are required to complete standardised training curricula administered by the Department of Health Service Support, Ministry of Public Health. Upon registration, new VHVs complete a foundational curriculum covering primary health care, health promotion, disease prevention, and basic community health reporting (1). Beyond initial training, a series of continuing education curricula have been progressively introduced, including a refresher training curriculum for active VHVs, a specialist VHV curriculum covering ten health domains (communicable disease surveillance, prevention and control; health promotion and non-communicable disease prevention; community mental health promotion; drug problem prevention; primary health care centre services and health security; consumer health protection; local health wisdom promotion; HIV/AIDS prevention and management; community health management; and maternal and child health) (1), and the VHV 4.0 Competency Standards Curriculum, which incorporates digital health literacy and smartphone-based disease reporting in alignment with Thailand’s national digital transformation policy (5). Despite the availability of these structured pathways, training coverage among VHVs remains incomplete, as a considerable proportion have not completed all available curricula, particularly in rural areas (8, 15). Consequently, observed competency levels among VHVs should be interpreted in the context of a workforce with varied, rather than absent, prior training exposure.

To address these gaps, this study applied Participatory Action-Oriented Research (PAOR: Plan–Act–Observe–Reflect) (16) as the guiding methodological framework. The PAOR cycle facilitated iterative, community-engaged processes in which VHVs and relevant stakeholders collaboratively assessed local disease control challenges, developed contextually appropriate competency development strategies, and evaluated implementation outcomes. This approach enabled the integration of emerging competency priorities—including digital literacy and risk communication—into a structured training model grounded in local practice realities. The study aimed to: (1) assess the level of functional competency of VHVs in Chaiyaphum Province in communicable disease prevention and control; (2) develop a contextually appropriate competency development model through the PAOR process; and (3) evaluate the effectiveness of the resulting model on VHV competency outcomes, with implications for sustainable capacity development at the district and subdistrict levels.

## 2 Methods

### 2.1 Research design

This study employed a mixed-methods Multiphase (Multi-sequenced) Design, integrating quantitative and qualitative data across three phases to mutually validate and expand findings. The study was guided by two complementary theoretical frameworks: the competency-based training framework, which conceptualises competency as the integrated application of knowledge, skills, and attitudes (KSAs) within defined professional roles (9, 10), and the community health workforce capacity development framework, which provides a systems-level model encompassing competency-based training, supportive supervision, health system integration, and performance monitoring and evaluation (11, 13). These frameworks were operationalised through the Participatory Action-Oriented Research (PAOR: Plan–Act–Observe–Reflect) cycle (16), ensuring that model development addressed both individual VHV skill acquisition and the organisational conditions required to sustain competency gains in practice. Data collection spanned March 2022 to July 2024, across three clearly demarcated phases:

- Phase 1 (March 2022–May 2023): a descriptive survey study to assess VHV competency levels and contextual factors across Chaiyaphum Province.
- Phase 2 (June 2023–March 2024): a participatory action research phase in which 34 stakeholders collaboratively designed the training programme structure, content, and delivery format based on Phase 1 findings, guided by the PAOR cycle.
- Phase 3 (April 2024–August 2024): a model evaluation phase assessing pre- and post-intervention competency changes among 33 VHVs in the target community. This phase used a one-group pretest–posttest design to evaluate the SMART VHV Plus Model among a separate intervention group. Competency was assessed before and after programme implementation using an integrated competency performance assessment.

### 2.2 Study setting

This study was conducted in Chaiyaphum Province, located in the upper northeastern (Isan) region of Thailand, comprising 16 districts, 124 subdistricts, and 1,547 villages, with a population of approximately 1.12 million (17). The economy is predominantly agricultural, with a high proportion of residents aged 50 years and older. Health services are delivered through one provincial hospital, 16 district hospitals, and 204 subdistrict health-promoting hospitals, supported by 24,247 registered VHVs as of 2022 (18). Digital infrastructure is unevenly distributed: urban areas have relatively reliable connectivity, while rural villages—where most VHVs operate—experience limited internet access and lower digital literacy, particularly among older residents. During the COVID-19 pandemic (2020–2022), VHVs assumed expanded frontline roles including contact tracing, home isolation monitoring, and community health communication, exposing substantive gaps in digital reporting, risk communication, and systematic data management that provided the primary impetus for this study.

### 2.3 Population and sample

The population comprised all registered Village Health Volunteers (VHVs) under Ministry of Public Health jurisdiction in Chaiyaphum Province, totaling 24,247 individuals. The study was conducted in three phases with distinct sampling strategies applied to each.

#### 2.3.1 Phase 1—quantitative sample (*n* = 416)

**Figure 1:**
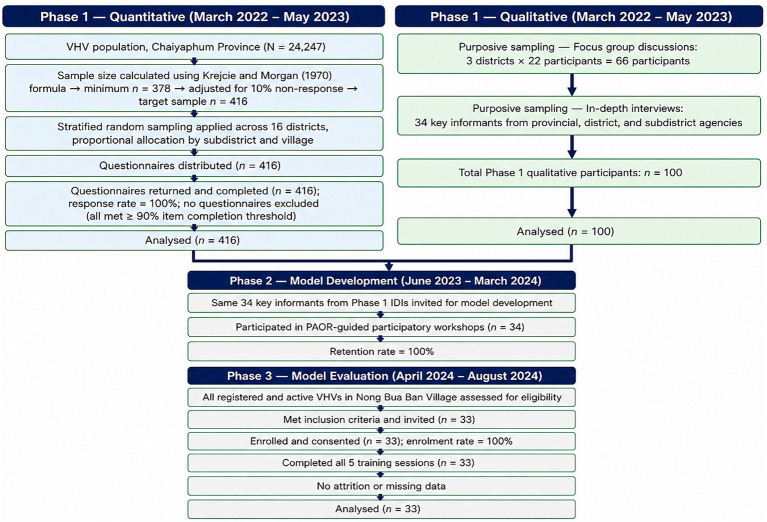
CONSORT-style participant flow diagram illustrating recruitment, sampling, and retention across the three study phases. Phase 1 comprised parallel quantitative (*n* = 416) and qualitative (*n* = 100) data collection. Phase 2 involved participatory model development with 34 stakeholders. Phase 3 evaluated the SMART VHV Plus Model with 33 VHVs in Nong Bua Ban Village, Chaiyaphum Province, Thailand.

The Phase 1 quantitative sample size was determined using the finite population formula of Krejcie and Morgan (19), applied to the total registered VHV population in Chaiyaphum Province (*N* = 24,247). The formula incorporates a chi-square value of 3.841 (df = 1, corresponding to a 95% confidence level), a population proportion of *p* = 0.50 (the conservative maximum variance estimate yielding the largest required sample), and an acceptable margin of error of *e* = 0.05. Substituting these parameters yielded a minimum required sample of *n* = 378. To account for anticipated non-response and incomplete questionnaires, 10% was added to this Figure (378 × 1.10 = 415.8), resulting in a final target sample of *n* = 416. Of the 416 questionnaires distributed, all 416 were returned and completed, yielding a response rate of 100%. Participant flow across all three phases is summarised in Figure 1. No questionnaires were excluded on the basis of missing data, as all returned instruments met the completeness threshold of ≥90% items completion.

Participants were selected using multi-stage stratified random sampling, with the province’s official VHV registry serving as the sampling frame. The population was stratified sequentially by district (16 districts), subdistrict, and village. Within each stratum, sample allocation was proportional to the number of registered VHVs, ensuring representative coverage across all administrative units. Simple random sampling was then applied within each village stratum to select individual participants.

#### 2.3.2 Phase 1—qualitative sample (*n* = 100)

Purposive sampling was used separately for qualitative data collection. Focus group discussions (FGDs) were conducted in three target districts—Khon San, Kaeng Khro, and Chaturat—with one FGD group per district comprising 22 participants each (3 FGD groups; total: *n* = 66). In-depth interviews (IDIs) were conducted with 34 key informants from agencies supporting VHV operations, including provincial public health officers (communicable disease control and primary health care), district public health officers, subdistrict health-promoting hospital directors, provincial and district VHV presidents, VHV excellence awardees, subdistrict administrative officers, and disease surveillance officers at provincial, district, and subdistrict levels (Phase 1 qualitative total: *n* = 100). Topics covered: communicable disease risk factors and the existing surveillance system in Chaiyaphum Province, and prior VHV competency development experiences. Inclusion criteria (Phase 1 qualitative): (1) involved in VHV operations at any level; (2) voluntary participation; (3) able to communicate; (4) no health conditions precluding participation. Exclusion criteria: (1) relocated outside Chaiyaphum Province prior to data collection; (2) incomplete interview data.

#### 2.3.3 Phase 2—stakeholder sample (*n* = 34)

The same 34 key informants from Phase 1 IDIs participated in the PAOR-guided participatory model development workshops. The study site—Nong Bua Ban Village, Nong Bua Ban Subdistrict, Chaturat District—was purposively selected for its strong community infrastructure, active VHV network, and direct COVID-19 outbreak experience, providing both enabling conditions and representative challenges for community-based action research.

#### 2.3.4 Phase 3—evaluation sample (*n* = 33)

All 33 registered and active VHVs in Nong Bua Ban Village meeting inclusion criteria were invited (census approach within the purposively selected site), and all agreed to participate (enrolment rate = 100%). Inclusion criteria: (1) residing in target area for ≥1 year; (2) voluntary participation with written consent; (3) able to communicate and attend training; (4) no health conditions precluding participation. Exclusion criteria: (1) unable to complete all five training sessions; (2) incomplete pre- or post-assessment data.

### 2.4 Research instruments and operational definitions

Two distinct quantitative instruments were developed and employed across the study phases, each designed to serve a different measurement purpose: a self-reported competency questionnaire for Phase 1 baseline assessment, and an objective performance test for Phase 3 intervention evaluation. These instruments are not directly comparable, as they measure different dimensions of competency using different scaling approaches.

The Phase 1 quantitative instrument comprised a structured questionnaire developed *de novo* by the research team through systematic review of relevant theoretical frameworks, national VHV competency standards established by the Department of Health Service Support (1), and prior empirical studies on VHV competency assessment in Thailand and comparable settings (6, 8, 15, 20). No pre-existing validated instrument directly applicable to this study’s scope and context was identified in the literature; the instrument was therefore constructed to reflect the specific competency domains, role functions, and local epidemiological priorities of VHVs in Chaiyaphum Province. The questionnaire comprised three sections relevant to competency assessment:

- *Section 1*: personal and occupational characteristics (7 items; checklist and open-ended format), covering sex, age, marital status, education level, occupation, monthly income, and years of VHV service.
- *Section 2*: VHV-specific role characteristics (6 items), covering households under care, population responsible for, prior training history across four standard curricula, disease outbreak experience, and budget allocation for disease control.
- *Section 3*: self-reported competency assessment across three domains, each measured on a five-point Likert scale (1 = lowest, 5 = highest): knowledge (epidemiological principles and disease prevention), skills (surveillance, data recording, and community health education), and practices (frequency and quality of health-promoting behaviours). Domain scores represent mean item scores with a possible range of 1.00–5.00, classified per Best (21) as low (1.00–2.33), moderate (2.34–3.66), or high (3.67–5.00).

A separate performance-based competency test was developed for Phase 3 to measure observable competency gains following the intervention, rather than self-perceived competency levels. The test assessed integrated competency across three domains—knowledge (5 items aligned with SMART component S: Screen), skills (5 items aligned with SMART components M, A, and R: Management, Application, and Response), and practices (5 items aligned with SMART component T: Time)—covering communicable disease surveillance, digital reporting, risk communication, leadership, and community health planning. Each correct response was awarded one point, yielding a total possible score of 15 points, converted to a percentage score (number of correct responses ÷ 15 × 100; range: 0–100%), classified as high (≥80%; 13–15 items correct), moderate (60–79%; 9–12 items correct), or low (<60%; 0–8 items correct) per criterion-referenced evaluation guidelines (22). Although the instrument covered three competency domains, the limited number of items per domain (5 items each) was considered insufficient for robust domain-specific inferential analysis. Scores were therefore aggregated into a single composite percentage score for pre-post comparison, providing a more reliable and interpretable measure of overall integrated competency change. The full instruments for both phases, including all items, answer options, scoring keys, and administration procedures, are provided in Supplementary Tables 1, 2.

Content validity of the instrument was assessed by three subject-matter experts in public health, communicable disease control, and community public health, using the Item-Objective Congruence (IOC) method (23). IOC values for all retained items ranged from 0.6 to 1.0, exceeding the acceptable threshold of 0.50. The instrument was subsequently pilot-tested with 30 VHVs in Ban Chuan Subdistrict, Bamnet Narong District, Chaiyaphum Province—a comparable population not included in the main study—to assess reliability. Internal consistency was confirmed with Cronbach’s alpha = 0.87, exceeding the acceptable threshold of 0.70.

Qualitative data collection instruments comprised: (a) a semi-structured focus group discussion (FGD) guide covering VHV roles, barriers, and competency development needs in communicable disease prevention and control; and (b) a structured in-depth interview guide for key informants from supporting agencies.

For the purposes of this study, key constructs are operationally defined as follows. Competency refers to the integrated capacity to perform health-related tasks effectively, encompassing knowledge, skills, and practices. Knowledge refers to understanding of epidemiological principles, disease transmission, prevention strategies, and outbreak response protocols. Skills refers to the ability to apply knowledge in structured surveillance, data recording, and community health education activities. Practices refers to the frequency and quality of health-promoting behaviours performed in community roles. Digital literacy refers to the ability to access, evaluate, and apply health information using smartphones and digital reporting platforms. Risk communication refers to the real-time exchange of information between health authorities, VHVs, and community members to enable informed decision-making during health threats. Leadership refers to the capacity to initiate, coordinate, and sustain community health activities through motivation, planning, and collaborative problem-solving.

### 2.5 Qualitative data collection and analysis

In Phase 1, FGDs were conducted in three districts (Khon San, Kaeng Khro, Chaturat) with 22 participants per district (*n* = 66 total). Duration per FGD: approximately 60–90 min. Additionally, 34 key informants participated in IDIs (duration: approximately 45–60 min each). The characteristics and organisational affiliations of key informants are detailed in Supplementary Table 3. In Phase 2, participatory workshops and FGDs were conducted with the same 34 stakeholders. All qualitative sessions were conducted in Thai at community venues familiar to participants, audio-recorded with written consent, and transcribed verbatim.

Qualitative data were analysed using the content analysis framework of Hsieh and Shannon (24), involving systematic inductive coding, categorisation into themes, and interpretive synthesis. Two researchers (P. C. and S. L.) independently reviewed and coded a subset of transcripts, then met to compare interpretations, discuss discrepancies, and reach consensus on final theme definitions and coding decisions. This consensual process was applied iteratively throughout the analysis until full agreement was achieved between coders. Rather than computing formal inter-coder reliability statistics, trustworthiness was ensured through the consensual coding process, peer debriefing, and member checking, consistent with established practice in participatory and applied qualitative health research (25). Qualitative data management was conducted manually, with transcript data systematically organised into printed coding sheets categorised by theme, subtheme, and interpretive notation to facilitate iterative review and consensual coding between researchers. Trustworthiness was established in accordance with Nowell et al. (26) through four criteria: credibility, ensured via member checking with a subset of participants; transferability, supported through thick contextual description of the study site, community, and participant characteristics; dependability, maintained through an audit trail of analytical decisions; and confirmability, strengthened through ongoing peer debriefing between the research team members. Theoretical saturation was considered achieved when no new themes emerged across successive data collection sessions. With respect to researcher reflexivity, the principal investigator (P. C.) is a public health officer employed within the same district as the study site; while this positionality facilitated access and rapport with participants, the potential for social desirability bias was acknowledged and mitigated through the use of structured interview guides, independent coding, and member checking procedure.

### 2.6 Quantitative data analysis

All quantitative analyses were performed using IBM SPSS Statistics Version 26.0 (IBM Corp., Armonk, NY, USA).

For Phase 1 (*n* = 416), descriptive statistics were used to characterise VHV demographic and occupational profiles, and to summarise baseline competency levels across all three domains. Statistics reported include frequencies, percentages, means, and standard deviations. Competency domain scores, derived from five-point Likert-scale items, were classified into three levels using the criteria of Best (21). To examine factors associated with overall VHV competency classification in Phase 1, chi-square tests (*χ*^2^) were conducted for eight demographic variables: sex, age group, education level, primary occupation, years of VHV service, number of households under care, prior training history, and disease outbreak experience in the area. Given the highly skewed distribution of competency scores—with only 1.68% of participants (*n* = 7) classified at the low level—overall competency was dichotomised into high (score ≥3.67; *n* = 265, 63.70%) versus below high (score <3.67; *n* = 151, 36.30%) for the purposes of these analyses. This dichotomisation was adopted to ensure adequate cell counts for valid chi-square testing and to reflect the substantively meaningful distinction between participants achieving high competency and those who had not. All expected cell counts exceeded 5, confirming the appropriateness of the chi-square test for all analyses. Statistical significance was set at *α* = 0.05.

For Phase 3 (*n* = 33), inferential analysis was conducted to compare overall competency percentage scores before and after the intervention. Prior to hypothesis testing, the normality of difference scores was examined using the Shapiro–Wilk test, which is recommended for small samples (*n* < 50) and is more powerful than the Kolmogorov–Smirnov test in this context (27). The test confirmed no significant deviation from normality, supporting the use of the parametric paired-samples *t*-test. Although normality was confirmed, the Wilcoxon signed-rank test was additionally conducted as a non-parametric sensitivity analysis given the small sample size (*n* = 33). Results were consistent with the paired-samples *t*-test, supporting the robustness of the parametric findings.

The paired-samples *t*-test was then applied to determine whether the mean difference between pre- and post-intervention scores was statistically significant, with the significance threshold set at *α* = 0.05 (two-tailed). Effect size was calculated as Cohen’s *d*, defined as the mean difference divided by the standard deviation of the difference scores, to quantify the practical magnitude of observed change beyond statistical significance. Interpretation followed conventional benchmarks: *d* = 0.2 (small), *d* = 0.5 (medium), and *d* = 0.8 (large) (28). As the Phase 3 evaluation employed a single composite competency outcome measure, only one inferential test was conducted and multiple comparison correction was therefore not required. A post-hoc power analysis was subsequently conducted using G*Power version 3.1 to determine the achieved statistical power (1 − *β*) for the observed effect size at the given sample size and significance level, providing an assessment of the study’s capacity to detect the observed effect.

### 2.7 Ethical considerations

This study received ethical approval from the Human Research Ethics Committee of Mahasarakham University across two rounds corresponding to the study phases. The first approval (No. 400-412/2565) was granted on 13 December 2022 and remained valid through 12 December 2023, covering Phase 1 data collection. The second approval (No. 412-107/2567) was granted on 27 June 2024 and remains valid through 26 June 2025, covering Phases 2 and 3. All research procedures were conducted in full accordance with the ethical standards of the institutional review committee and applicable national regulations governing human subjects research. Prior to participation, all individuals provided written informed consent, and confidentiality of personal data was maintained throughout the study.

## 3 Results

### 3.1 Phase 1: community context and baseline assessment

Phase 1 was conducted from March 2022 to May 2023 and enrolled 416 VHVs across all 16 districts of Chaiyaphum Province. Participant flow through all three phases of the study, including sampling procedures, response rates, and retention, is presented in Figure 1. Demographic characteristics are presented in Table 1.

**Table 1:** Demographic and occupational characteristics (*n* = 416).

Characteristic	Category	*n*	%
Sex			
	Female	335	80.53
	Male	81	19.47
Age (mean = 53.33, SD = 9.20, Min = 23, Max = 82)			
	< 31 years	7	1.68
	31–40 years	29	6.97
	41–50 years	117	28.13
	51–60 years	179	43.03
	>60 years	84	20.19
Education			
	No formal education	2	0.48
	Primary school	151	36.30
	Lower secondary school	105	25.24
	Upper secondary school/vocational cert.	141	33.89
	Diploma (associate degree)	14	3.37
	Bachelor’s degree or higher	3	0.72
Primary occupation			
	Unemployed (no formal job)	8	1.92
	Agriculture (farmer)	307	73.80
	Trade or small business	16	3.85
	General laborer	78	18.75
	Government/State enterprise employee	7	1.68
Years as a VHV (mean = 15.99, SD = 8.66, Min = 1, Max = 44)			
	1–10 years	131	31.49
	11–20 years	188	45.19
	21–30 years	74	17.79
	31–40 years	21	5.05
	>40 years	2	0.48
Households under VHV care (mean = 9.88, SD = 2.79, Min = 4, Max = 24)			
	4–8 households	122	29.33
	9–13 households	259	62.26
	14–18 households	32	7.69
	19–23 households	2	0.48
	≥24 households	1	0.24
Ever attended a capacity-building training			
	Yes	304	73.08
	No	112	26.92
History of a disease outbreak in area			
	Yes	119	28.61
	No	297	71.39
Primary VHV responsibility			
	Disease surveillance	191	45.91
	Disease prevention	94	22.60
	Disease control	131	31.49

The majority of VHVs were female (80.53%), aged 51–60 years (43.03%; mean = 53.33, SD = 9.20), married (84.61%), with primary school education (36.30%), employed in agriculture (73.80%), and serving 11–20 years as VHVs (45.19%; mean = 15.99, SD = 8.66). Most were responsible for 9–13 households (62.26%) and 16–31 individuals (48.32%). Regarding disease context, outbreaks occurred in 28.61% of VHVs’ areas, predominantly COVID-19 (11.54%), hand-foot-and-mouth disease and diarrhoea (10.09%), and dengue fever (3.12%). The primary VHV responsibility was disease surveillance (45.91%), followed by disease control (31.49%) and disease prevention (22.60%). Of note, 26.92% had never received any capacity-building training, and disease control budgets were predominantly below 10,000 THB (83.65%), financed primarily through the National Health Security Office fund (87.3%).

Baseline self-reported competency results are presented in Table 2. Overall competency was rated at a high level (mean = 3.71, SD = 0.46), with practices the strongest domain (mean = 4.34, SD = 0.42) and knowledge the weakest (mean = 3.59, SD = 0.49; 17.79% at low level). Standard deviations across the three domains were comparable (knowledge SD = 0.49, skills SD = 0.45, practices SD = 0.42), indicating consistent distributional properties. The lower mean knowledge score relative to skills (mean = 3.60) and practices (mean = 4.34), despite similar dispersion, reflects a systematic gap in theoretical competency rather than heterogeneity in measurement properties, consistent with the tendency for community health workers to accumulate practical experience ahead of formalised knowledge in contexts where on-the-job learning predominates. The high practices score approaching the scale ceiling (mean = 4.34 of 5.00) further suggests that self-reported competency measures may underestimate training need in higher-order competency areas such as epidemiological reasoning and digital reporting.

**Table 2:** Baseline competency assessment by domain (*n* = 416).

Domain	Mean	SD	Min	Max	Low *n* (%)	Moderate *n* (%)	High *n* (%)	Level
Knowledge	3.59	0.49	1.33	5.00	74 (17.79)	99 (23.80)	243 (58.41)	Moderate
Skills	3.60	0.45	2.10	4.80	64 (15.38)	147 (35.34)	205 (49.28)	Moderate
Practices	4.34	0.42	1.93	5.00	14 (3.37)	152 (36.54)	250 (60.09)	High
Overall	3.71	0.46	1.93	5.00	7 (1.68)	144 (34.62)	265 (63.70)	High

Five-point Likert scale (1.00–5.00). Best (21) classification: ≤2.33 = low, 2.34–3.66 = moderate, ≥3.67 = high.

Chi-square analyses were conducted to examine associations between demographic and occupational characteristics and overall VHV competency classification (high versus below high; *n* = 265 and *n* = 151 respectively). No statistically significant associations were identified for any of the variables examined, including sex (*χ*^2^ = 0.809, df = 1, *p*-value = 0.369), age group (*χ*^2^ = 0.183, df = 1, *p*-value = 0.669), education level (*χ*^2^ = 3.034, df = 1, *p*-value = 0.082), primary occupation (*χ*^2^ = 0.313, df = 1, *p*-value = 0.576), years of VHV service (*χ*^2^ = 0.427, df = 1, *p*-value = 0.513), number of households under care (*χ*^2^ = 2.402, df = 1, *p*-value = 0.121), prior training history (*χ*^2^ = 1.007, df = 1, *p*-value = 0.316), and disease outbreak experience in the area (*χ*^2^ = 0.701, df = 1, *p*-value = 0.402). These findings suggest that overall competency level among VHVs in Chaiyaphum Province was not significantly differentiated by individual demographic or occupational characteristics, implying that competency gaps were distributed broadly across the workforce rather than concentrated in specific subgroups. This pattern reinforces the rationale for a province-wide, inclusive competency development intervention rather than a targeted subgroup approach.

Qualitative findings from Phase 1 focus group discussions (*n* = 66; 3 groups, 22 VHVs per group) and in-depth interviews (*n* = 34) identified five thematic competency gap domains:

- *Knowledge and situational assessment*: VHVs articulated limited confidence in epidemiological reasoning and difficulty interpreting surveillance data for situational analysis.
- *Data recording and management*: data recording practices remained predominantly paper-based and non-standardised, resulting in inconsistent reporting outcomes.
- *Digital literacy and technology use*: digital literacy barriers were pronounced among VHVs aged 50 years and older, who frequently relied on family members to complete online disease reports.
- *Risk communication and public education*: risk communication capacity was constrained by low confidence in conveying health information and in addressing community misinformation.
- *Leadership and teamwork*: leadership orientation was primarily reactive, with most VHVs awaiting directives from health service units rather than initiating community health activities independently.

Collectively, these qualitative findings revealed substantive capability limitations not fully reflected in the quantitative self-report scores, reinforcing the need for structured competency development intervention and providing the empirical foundation for the five components of the SMART VHV Plus Model.

### 3.2 Phase 2: development of the SMART VHV Plus Model

The SMART VHV Plus Model was developed through the PAOR cycle (June 2023–March 2024) with 34 stakeholders (including VHV leaders, public health officers, and local administrative personnel; see Section 3.3). The PAOR cycle was operationalised across four sequential stages. In the *Plan* stage, Phase 1 findings were presented to local stakeholders, and a competency development plan was jointly designed based on the knowledge, skills, and practice gaps identified in the baseline assessment. In the *Act* stage, five training programmes were implemented in accordance with the model components, covering participatory management, digital competency, advanced disease control, community health planning, and leadership and teamwork development. In the *Observe* stage, data were collected before and after each training session alongside qualitative observations and reflective feedback, to assess the extent to which each programme addressed VHV competency needs. In the *Reflect* stage, participatory review meetings were conducted among VHVs, key informants, and relevant stakeholders to share experiences, consolidate lessons learnt, and develop recommendations for model refinement, contributing to iterative improvement of the model for potential application in other settings.

**Figure 2:**
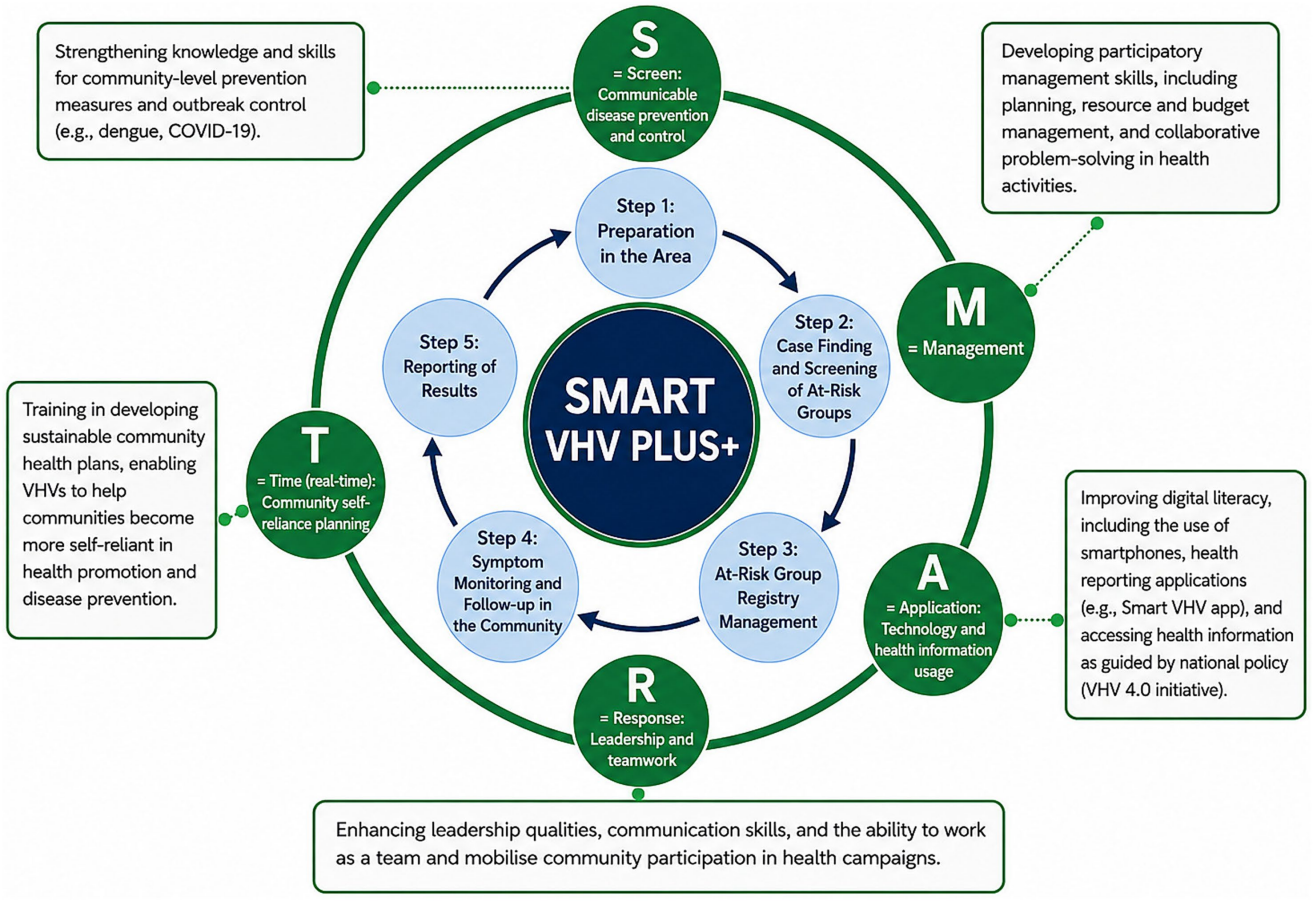
The SMART VHV Plus Model for competency development of Village Health Volunteers (VHVs) in community-based communicable disease surveillance, prevention, and control, Chaiyaphum Province, Thailand. The five outer nodes (S, M, A, R, T) represent the five competency development components of the model, each linked to a corresponding training programme and operational domain (shown in annotation boxes). The five inner nodes represent the sequential operational steps of the VHV disease surveillance and response workflow. The model integrates both competency development (outer structure) and operational practice (inner workflow) to reflect the functional context in which VHV competencies are applied.

The SMART acronym maps to five operational competency domains as follows: S (Screen) = communicable disease surveillance and at-risk group screening; M (Management) = participatory resource management and collaborative problem-solving; A (Application) = digital technology application and health information systems (aligned with national VHV 4.0 initiative); R (Response) = community-level outbreak response and risk communication; T (Time) = timely, self-reliant community health planning (Figure 2). The designation “Plus” signifies extension beyond foundational Ministry of Public Health VHV standards to incorporate three emerging domains not previously systematised: digital literacy, leadership, and community self-reliance planning (Supplementary Table 4). The SMART VHV Plus Model is an original framework developed within this study and does not extend a pre-existing model of the same name. The designation “Plus” was coined by the research team to signify the additive nature of the three emerging competency domains incorporated beyond the foundational Ministry of Public Health VHV competency standards, rather than to reference any prior published framework.

### 3.3 Phase 3: evaluation of the SMART VHV Plus Model

The Phase 3 evaluation focused on changes in integrated functional competency following implementation of the SMART VHV Plus Model. The competency assessment in this phase was designed to capture overall applied competency performance across the intervention domains rather than isolated domain-specific outcomes. This phase was conducted from April to August 2024 with 33 VHVs in Nong Bua Ban Village who completed all five training sessions. The integrated competency performance test (15 items; range: 0–100%) was administered immediately before the first training session (Programme 1: Advanced Disease Prevention and Control Training) as the overall pre-intervention baseline, and immediately following the final session (Programme 5: Community Health Plan Development Programme) as the post-intervention outcome. Phase 3 participants were predominantly female (83.20%), aged 46–55 years (35.10%; mean = 55.18, SD = 10.48, range 28–65), with upper secondary education (39.20%) and agricultural occupation (78.02%).

Prior to statistical analysis, the Shapiro–Wilk test confirmed normality of difference scores (no significant deviation from normality), supporting use of the paired-samples t-test (Table 3).

**Table 3:** Pre- and post-intervention competency scores (*n* = 33).

Variable	*n*	Mean (%)	SD	SE	95% CI		*p*-value
					Lower	Upper	
Pre-intervention (moderate level)	33	86.14	7.653	1.332	83.43	88.86	<0.05^*^
Post-intervention (high level)	33	98.16	1.951	0.340	97.47	98.85	
Difference	33	12.02	6.140	1.068	9.84	14.20	

Scores represent percentage of correct responses on the integrated competency performance test (range: 0–100%); classified as high (≥80%), moderate (60–79%), or low (<60%) per Bloom (22). Normality of difference scores was confirmed by the Shapiro–Wilk test; the Wilcoxon signed-rank test yielded consistent results. Effect size: Cohen’s *d* = 1.96; 95%CI of mean difference: 9.84–14.20. Domain-specific pre-post scores are not reported separately. Although the instrument assessed competency across three domains (knowledge, skills, and practices; 5 items each), the limited number of items per domain was considered insufficient for valid domain-specific inferential analysis; scores were therefore aggregated into a single composite percentage score for pre-post comparison. (see Section 3.4 and Supplementary Table 2). ^*^p-value < 0.05.

Prior to the training programme, participants demonstrated a moderate overall competency level (mean = 86.14%, SD = 7.65, 95% CI: 83.43–88.86). Following completion of all five SMART VHV Plus Model training sessions, mean competency increased to the high level (mean = 98.16%, SD = 1.95, 95% CI: 97.47–98.85), representing a mean difference of 12.02 percentage points (95% CI: 9.84–14.20, *p* < 0.05). Effect size was calculated as Cohen’s *d* = 1.96, with Hedges’ *g* = 1.91 applied as a bias-corrected alternative appropriate for small samples (*n* = 33). Both values indicate a large effect per conventional benchmarks (28), consistent with the concentrated post-intervention score distribution (SD = 1.95) reflecting high and uniform competency attainment following the training programme. The large effect size should be interpreted in the context of the one-group design, wherein the absence of a control group means that the full magnitude of the effect cannot be attributed exclusively to the intervention. Post-hoc power analysis (G*Power version 3.1) confirmed that the achieved statistical power was 1 − *β* > 0.999, indicating that the sample was more than adequate to detect an effect of the observed magnitude.

## 4 Discussion

### 4.1 Principal findings

This study yielded three principal findings. First, baseline self-reported competency among VHVs in Chaiyaphum Province was overall high (mean = 3.71, SD = 0.46), yet with important heterogeneity: knowledge scores showed the widest distribution (SD = 0.49) and the highest proportion of low-rated participants (17.79%), while practices scores approached the scale ceiling (mean = 4.34). Second, qualitative data provided convergent evidence of substantive capability gaps in epidemiological reasoning, digital literacy, and risk communication not fully captured by quantitative self-report. Third, following implementation of the SMART VHV Plus Model (Phase 3, *n* = 33), participants demonstrated a statistically significant improvement in integrated overall competency scores, increasing from 86.14 to 98.16% (*p*-value <0.05). These findings suggest that the participatory and multi-component structure of the intervention may contribute to strengthening multiple competency dimensions relevant to communicable disease prevention and control. Chi-square analyses of Phase 1 data found no statistically significant associations between overall competency classification and any of the demographic or occupational variables examined, including sex, age, education, occupation, years of service, training history, or disease outbreak experience (*p*-value >0.05); rather than indicating an absence of competency development need, this finding suggests that gaps were broadly distributed across the VHV workforce regardless of individual background characteristics, supporting the rationale for a universal rather than targeted intervention design. However, because the Phase 3 evaluation prioritised integrated functional competency assessment rather than separate domain-specific outcome measures, the relative contribution of individual competency domains could not be independently determined.

### 4.2 Alignment with global and national competency frameworks

The competency domains incorporated within the SMART VHV Plus Model align with WHO and Community Health Worker Core Competency (C3) framework guidance, which emphasises competency-based training, clearly defined roles, supportive supervision, and health system integration (10, 11). The Screen and Response components correspond to competencies related to disease surveillance, outbreak preparedness, and community-level response emphasised in WHO community health workforce guidance (11). The Application component reflects increasing international and national emphasis on digital literacy and smartphone-based health reporting, including alignment with Thailand’s VHV 4.0 initiative. Similar competency priorities have been reported in community health worker programmes across low- and middle-income countries. India’s Accredited Social Health Activists (ASHA) programme has demonstrated that motivation, supervision, and structured competency-based training are critical determinants of CHW performance (29). Ethiopia’s Health Extension Worker system and Pakistan’s Lady Health Worker programme similarly highlight the importance of system-level support, clearly defined roles, and ongoing supervision in sustaining community health workforce capacity (14). These comparative examples underscore that the competency gaps identified among VHVs in this study—particularly in digital literacy, data management, and risk communication—reflect broader challenges common to CHW programmes in LMICs, and that structured, contextually tailored interventions such as the SMART VHV Plus Model may offer transferable lessons for similar workforce development initiatives in the region.

### 4.3 Reconciling baseline findings with identified gaps

The apparent discrepancy between high aggregate quantitative competency scores and qualitatively identified substantive gaps warrants explicit interpretation. Three mechanisms are proposed. First, social desirability bias is a well-documented limitation of self-reported competency measures in CHW populations, particularly where perceived adequacy may be implicitly expected (10). VHVs who reported high practice scores may have rated routine behaviours favourably while underestimating gaps in higher-order capabilities. Second, ceiling effects limit the sensitivity of the practices scale (mean = 4.34 of 5.00) to detect meaningful variation or improvement. Third, self-report and qualitative data capture distinct competency dimensions: self-report reflects perceived confidence and behavioural frequency, while reflective qualitative accounts reveal limitations in emerging areas—epidemiological reasoning, digital reporting, risk communication—not historically emphasised in standard Ministry of Public Health VHV training curricula. Furthermore, the comparable standard deviations across all three competency domains (knowledge SD = 0.49, skills SD = 0.45, practices SD = 0.42) indicate that the observed gap between knowledge and practices reflects a systematic difference in mean competency level rather than differential measurement sensitivity, reinforcing the interpretation that the instrument’s ceiling effects—rather than measurement heterogeneity—account for the discrepancy between high self-reported scores and qualitatively identified gaps. These findings argue for composite multi-method competency assessment over exclusive reliance on self-report instruments. The integrated competency assessment approach used in Phase 3 was conceptually aligned with the competency-based framework underpinning the SMART VHV Plus Model, in which competency was defined as the combined application of knowledge, practical skills, behavioural practices, communication capacity, and contextual decision-making in real-world community health settings. Accordingly, the evaluation prioritised overall functional competency performance rather than isolated technical domains alone.

### 4.4 Effectiveness and comparison with previous studies

The observed improvement in overall competency scores following implementation of the SMART VHV Plus Model is consistent with previous evidence indicating that competency-based training can strengthen community health worker performance across diverse settings (10, 14). At the national level, competency development programmes have been associated with improved VHV knowledge and health promotion practices in non-communicable disease prevention (30), while improved health literacy has been linked to enhanced disease prevention behaviours among VHVs (31). International studies have also demonstrated the potential of blended and multi-component learning approaches to improve digital competency and reporting practices among community health workers in low- and middle-income countries (32). Collectively, these findings support the relevance of integrated competency development approaches in community-based disease prevention and control.

However, the findings should be interpreted with caution. The one-group pretest–posttest design without a comparison group limits causal attribution of the observed improvements to the intervention alone (33). In addition, the relatively high pre-intervention competency scores (86.14%) may indicate limited instrument sensitivity to detect change in a generally experienced VHV population, consistent with broader concerns regarding competency measurement in community health workforce research (10, 34).

Beyond statistical significance, the practical meaningfulness of the observed improvement warrants consideration. A mean competency score increase of 12.02 percentage points—from 86.14% (moderate) to 98.16% (high)—represents a clinically meaningful threshold shift in competency classification, moving the entire group from below to above the high competency benchmark (≥80%). The large effect size (Cohen’s *d* = 1.96) further supports the practical magnitude of this change. In the context of community-based disease surveillance, this level of competency gain may translate into more accurate case detection, timelier digital reporting, and more confident risk communication with community members—outcomes with direct implications for outbreak containment at the community level. However, without long-term follow-up or objective performance data (e.g., disease reporting accuracy rates), the extent to which competency gains translate into sustained behavioural change in practice remains to be established.

### 4.5 Challenges and considerations for scale-up

Communities with a higher proportion of older VHVs or limited digital infrastructure may experience slower implementation of technology-based components within the SMART VHV Plus Model, consistent with evidence identifying age, education, and digital access as important determinants of digital health literacy adoption in Thailand (35). In addition, implementation capacity may vary across provincial and local health systems due to differences in governance structures, resource availability, organisational support, and workforce readiness. These contextual factors may influence implementation fidelity, sustainability, and scalability of the model across settings. Accordingly, successful scale-up may require coordinated cross-sector collaboration, governance readiness, and sustained system-level support to ensure equitable implementation (12, 36).

Sensitivity analyses were considered but could not be conducted, as the one-group design and small sample size (*n* = 33) did not permit meaningful subgroup or alternative model specification analyses. Future studies with larger samples and comparison groups would enable more robust sensitivity testing.

The findings of this study carry several specific implications for policy and practice. At the national level, the five competency domains of the SMART VHV Plus Model—particularly digital literacy and risk communication—could be considered for formal integration into the existing national VHV specialist curriculum administered by the Department of Health Service Support, Ministry of Public Health, thereby institutionalising these emerging competencies within the standard VHV development pathway. At the provincial level, Chaiyaphum Provincial Public Health Office and counterpart offices in similar rural provinces could adopt the PAOR-guided model development process as a replicable framework for contextually tailored VHV capacity building, rather than relying solely on centrally designed training packages. At the district level, subdistrict health-promoting hospitals—which serve as the primary operational interface between VHVs and the formal health system—could utilise the structured observation checklists and feedback mechanisms developed in this study as ongoing fidelity monitoring tools. Finally, given that 26.92% of sampled VHVs had never received any capacity-building training, targeted outreach to this subgroup should be prioritised in any scale-up initiative to ensure equitable competency development across the VHV workforce.

### 4.6 Strengths, limitations, and future research

Strengths of this study include the use of a rigorous Multiphase mixed-methods design, the large baseline sample (*n* = 416) providing a robust provincial-level competency profile, the PAOR-guided participatory model development ensuring contextual relevance, and the model’s five components addressing the specific contemporary competency gaps identified through both quantitative and qualitative methods.

The internal validity of pre-post findings is subject to several well-recognised threats inherent to the one-group pretest-posttest design. History effects cannot be excluded, as concurrent public health activities or district-level interventions during the study period may have independently contributed to observed competency gains. Maturation effects are plausible given VHVs’ continued routine health work throughout the intervention, while testing effects may have occurred if pre-test exposure sensitised participants to assessed content domains. Regression to the mean remains a consideration given the purposive nature of site selection, and Hawthorne effects may have influenced both training engagement and post-test performance. A further limitation concerns the aggregation of competency domains into a single composite score: although this reflects the integrated nature of community health practice, it precludes identification of which specific domains drove the observed improvements. Collectively, these threats preclude causal attribution of observed gains to the SMART VHV Plus Model alone (33). With respect to external validity, findings are limited to VHVs in a single rural village with strong community infrastructure and prior COVID-19 experience, and cannot be directly generalised to the broader Chaiyaphum Province VHV population (*N* = 24,247) or to other settings. The absence of long-term follow-up further leaves questions about the sustainability of competency gains unanswered. Future research should employ quasi-experimental designs with matched comparison groups or stepped-wedge cluster-randomised trials, incorporate domain-specific outcome measures and longitudinal follow-up to examine differential competency development and sustainability, and extend to larger, more diverse, and cross-regional samples.

## Data Availability

The raw data supporting the conclusions of this article will be made available by the authors, without undue reservation.
