# Supplementary material for: A model for developing the competencies of Village Health Volunteers in the prevention and control of communicable diseases at the community level: a case study of Chaiyaphum Province, Thailand
Source: Front Public Health. 2026 Jun 25;14:1781727. doi: 10.3389/fpubh.2026.1781727 (PMC13346076; doi:10.3389/fpubh.2026.1781727)
Supplement: Supplementary file 1 [file Supplementary_file_1.DOCX]

Supplementary Material

# Supplementary Figures and Tables

**1.1 Supplementary Tables**

**Supplementary Table 1.** Questionnaire for assessment of Village Health Volunteers’ Competencies in Communicable Disease Prevention and Control (Phase 1).

| **Questions** | **Answers** | | | | |
| --- | --- | --- | --- | --- | --- |
| **Section 1: Personal Characteristics** | | | | | |
| Sex | ( ) Male ( ) Female | | | | |
| Age | ............................. years | | | | |
| Educational Attainment | ( ) No formal education ( ) Primary education ( ) Lower secondary education ( ) Upper secondary education/Vocational certificate ( ) Diploma/Associate degree ( ) Bachelor’s degree or higher | | | | |
| Occupation | ( ) Unemployed ( ) Agriculture ( ) Trade/Private business ( ) General labor ( ) Government officer/State enterprise employee ( ) Other (please specify) ..................................... | | | | |
| Duration of Service as a Village Health Volunteer (VHV) | ............................. years | | | | |
| **Section 2: Existing Competencies of VHVs** | | | | | |
| Number of households under your responsibility | ............................. households | | | | |
| Number of populations under your responsibility | ............................. persons | | | | |
| Budget allocation for communicable disease prevention and control in the community, Fiscal Year 2022 | ............................. Baht | | | | |
| Have you ever attended competency development training? | ( ) Yes ( ) No | | | | |
| Has a communicable disease outbreak occurred in your area? | ( ) Yes ( ) No | | | | |
| Suggestions regarding competency development of VHVs in the four domains of surveillance, prevention, and control of communicable diseases in communities (How should public health agencies enhance VHV competencies?) | ( ) Knowledge: ____________________________  ( ) Skills: _________________________________  ( ) Abilities/Capabilities: ____________________  ( ) Other suggestions: ______________________ | | | | |
| **Section 3: Self-Assessment of Knowledge, Skills, and Practices in Communicable Disease Prevention and Control** | | | | | |
| **Questions** | **Answers** | | | | |
|  | **Lowest** | **Low** | **Moderate** | **High** | **Highest** |
| 1. I can organize community meetings to identify communicable disease problems that are consistent with the actual health situations in my area. | 1 | 2 | 3 | 4 | 5 |
| 2. I can maintain records of population movement (inbound and outbound registration) in the community to help reduce the risk of communicable disease outbreaks. | 1 | 2 | 3 | 4 | 5 |
| 3. I can regularly analyze disease situation data in my area and detect communicable disease outbreaks in a timely manner. | 1 | 2 | 3 | 4 | 5 |
| 4. I can organize health promotion campaigns and develop public communication materials for communicable disease prevention within the community. | 1 | 2 | 3 | 4 | 5 |
| 5. I can make appropriate decisions regarding the procurement of materials and equipment necessary for communicable disease prevention and control within my area of responsibility. | 1 | 2 | 3 | 4 | 5 |
| 6. I possess the knowledge and ability to implement dengue prevention and control measures appropriately, particularly in eliminating mosquito breeding sites. | 1 | 2 | 3 | 4 | 5 |
| 7. I can promote community participation in communicable disease prevention and control activities to achieve sustainable problem-solving outcomes. | 1 | 2 | 3 | 4 | 5 |
| 8. I actively participate in communicable disease prevention and control operations in collaboration with public health personnel. | 1 | 2 | 3 | 4 | 5 |
| 9. I can recognize unusual events or risk factors associated with communicable diseases and promptly report or coordinate responses appropriately. | 1 | 2 | 3 | 4 | 5 |
| 10. I am competent in conducting surveillance, prevention, and control activities for communicable diseases within the community in a timely and effective manner. | 1 | 2 | 3 | 4 | 5 |
| 11. I possess knowledge and understanding regarding surveillance events, including disease occurrence and risk factors that may adversely affect public health. | 1 | 2 | 3 | 4 | 5 |
| 12. I can implement communicable disease control measures that emphasize primary prevention, including health promotion and hygiene improvement within the community. | 1 | 2 | 3 | 4 | 5 |
| 13. I can report communicable disease events and related information through both paper-based reporting systems and online platforms, such as mobile applications or Line groups. | 1 | 2 | 3 | 4 | 5 |
| 14. I can participate effectively in online meetings or training sessions through digital platforms such as Google Meet or Zoom. | 1 | 2 | 3 | 4 | 5 |
| 15. I can submit operational reports through online systems or digital applications, such as the Smart VHV application. | 1 | 2 | 3 | 4 | 5 |

**Note:** The original questionnaire was developed in the Thai language and used for data collection in the study setting. The English version presented in this supplementary material was translated for academic publication purposes. Translation was undertaken with attention to conceptual equivalence, contextual appropriateness, and consistency with the original instrument. Minor editorial revisions were applied to enhance clarity and readability in English without altering the substantive meaning of the questionnaire items.

**Supplementary Table 2.** Integrated Competency Performance Test (Phase 3).

| **SMART VHV Plus Model \| Communicable Disease Prevention and Control** | |
| --- | --- |
| **Instructions:** Select the best answer for each question. Each correct answer = 1 point. Total score = 15 points, converted to percentage (score ÷ 15 × 100). | |
| **Domain 1: Knowledge (5 items)**  **SMART Component: S — Screen (Communicable Disease Surveillance)** | |
| **Item 1** A VHV notices that three households in the same street have members with fever and rash within one week. What is the most appropriate first action? | A) Wait to see if more cases emerge before taking action  B) Report to the subdistrict health-promoting hospital immediately and begin case-finding in nearby households  C) Advise affected families to visit the district hospital independently  D) Distribute paracetamol to all affected households |
| **✅ Answer: B** *Rationale: Clustering of cases within a short timeframe and geographic area constitutes an outbreak signal requiring immediate notification and active case-finding, consistent with the 5-step VHV outbreak response protocol* | |
| **Item 2** Which of the following best describes the primary route of transmission of dengue fever? | A) Contaminated food and water  B) Direct contact with an infected person's blood  C) Bite of an infected Aedes mosquito  D) Airborne droplets from coughing or sneezing |
| **✅ Answer: C** *Rationale: Dengue fever is a vector-borne disease transmitted exclusively through the bite of an infected Aedes aegypti or Aedes albopictus mosquito.* | |
| **Item 3** In the context of the VHV 5-step outbreak response protocol, which step involves creating and maintaining a list of individuals at risk of infection in the community? | A) Step 1: Preparation in the Area  B) Step 2: Case Finding and Screening of At-Risk Groups  C) Step 3: At-Risk Group Registry Management  D) Step 4: Symptom Monitoring and Follow-up in the Community |
| **✅ Answer: C** *Rationale: At-Risk Group Registry Management (Step 3) involves systematic documentation and maintenance of a registry of individuals identified as at risk, enabling targeted monitoring and follow-up.* | |
| **Item 4** A VHV is conducting disease surveillance in the community. Which of the following criteria most accurately identifies a household as "at risk" for dengue fever? | A) The household has a member who works outside the village  B) The household has stagnant water containers that could serve as mosquito breeding sites  C) The household is located more than 500 metres from the health-promoting hospital  D) The household has elderly members aged over 60 years |
| **✅ Answer: B** *Rationale: Stagnant water containers are the primary breeding sites for Aedes mosquitoes, the vector of dengue fever. Identifying and eliminating breeding sites is the core of dengue surveillance and prevention.* | |
| **Item 5** Which of the following best describes the role of VHVs within Thailand's Surveillance and Rapid Response Team (SRRT) system? | A) VHVs lead all SRRT investigations independently without involvement of health officers  B) VHVs serve as community-level frontline members who report suspected cases and support SRRT investigations  C) VHVs are responsible only for distributing medication during outbreaks  D) VHVs participate in SRRT only when specifically invited by the district hospital |
| **✅ Answer: B** *Rationale: VHVs function as the community-level frontline of the SRRT system, responsible for early case detection, reporting, and supporting investigations led by public health officers.* | |
| **Domain 2: Skills (5 items)**  **SMART Components: M — Management, A — Application, R — Response** | |
| **Item 6** A VHV needs to submit a disease surveillance report to the subdistrict health-promoting hospital using the Smart VHV application. Which sequence of steps is correct? | A) Open application → select "Report Disease" → enter case details → submit → confirm submission received  B) Call the health officer first → enter data → submit → close application  C) Open application → take a photo of the patient → send via LINE application → log out  D) Submit a paper report first → then enter the same data into the application the following day |
| **✅ Answer: A** *Rationale: The correct procedure for digital disease reporting via the Smart VHV application follows a standardised sequence: open, select report type, enter details, submit, and confirm receipt — ensuring timely and accurate data entry.* | |
| **Item 7** A VHV is planning a community health activity to prevent dengue fever but has a limited budget of 3,000 THB from the local administrative organisation. Which approach best demonstrates participatory resource management? | A) Spend the entire budget on purchasing larvicide without consulting community members  B) Consult with community members to identify locally available resources and co-design a cost-effective prevention plan  C) Request additional funds from the district hospital before planning any activity  D) Cancel the activity until a larger budget is available |
| **✅ Answer: B** *Rationale: Participatory resource management involves engaging community members in identifying local resources and co-designing solutions, maximising impact within available budget constraints.* | |
| **Item 8** A VHV receives a message via social media claiming that drinking herbal tea can cure COVID-19. What is the most appropriate response? | A) Forward the message to all contacts to inform the community  B) Ignore the message as it is not the VHV's responsibility  C) Verify the information against credible sources (Ministry of Public Health website) before communicating accurate information to community members  D) Report the message to the police immediately |
| **✅ Answer: C** *Rationale: Risk communication competency includes the ability to identify misinformation, verify claims against authoritative sources, and deliver accurate health information to the community — a core VHV responsibility.* | |
| **Item 9** During a community meeting to plan a disease prevention campaign, VHVs disagree about which activities to prioritise. Which leadership approach, based on the Four-Directions leadership theory, is most appropriate for this situation? | A) Directing — the VHV leader makes all decisions independently to save time  B) Coaching — the VHV leader guides discussion, explains reasoning, and builds consensus among team members  C) Delegating — the VHV leader assigns all decisions to individual members without discussion  D) Withdrawing — the VHV leader steps back and allows the disagreement to resolve itself |
| **✅ Answer: B** *Rationale: When team members have varying levels of experience and confidence, a coaching style — combining guidance with participatory discussion — is most appropriate for building consensus and developing team capacity.* | |
| **Item 10** A VHV identifies that a community member who has been in contact with a confirmed COVID-19 case is not monitoring their symptoms. What is the correct action under the 5-step VHV outbreak response protocol? | A) Report the case to the village headman only  B) Conduct Step 4: Symptom Monitoring and Follow-up — visit the individual daily, monitor for symptoms, and report any changes to the subdistrict health-promoting hospital  C) Advise the individual to visit the hospital only if symptoms develop  D) Record the individual's name in a notebook and review after two weeks |
| **✅ Answer: B** *Rationale: Step 4 of the VHV outbreak response protocol requires active, daily symptom monitoring and follow-up of at-risk individuals, with immediate reporting of any symptom development to health authorities.* | |
| **Domain 3: Practices (5 items)**  **SMART Component: T — Time (Community Health Planning)** | |
| **Item 11** A VHV is developing a community health plan for communicable disease prevention. Which of the following components is essential for the plan to be considered complete and actionable? | A) A list of diseases only, without specifying activities or resources  B) Situation analysis, objectives, planned activities, responsible persons, timeline, budget, and evaluation indicators  C) A budget request submitted to the district hospital without community input  D) A copy of the previous year's plan with the date updated |
| **✅ Answer: B** *Rationale: A complete and actionable community health plan requires all core components: situation analysis, clear objectives, specific activities, assigned responsibilities, a realistic timeline, budget allocation, and measurable evaluation indicators.* | |
| **Item 12** After implementing a dengue prevention campaign, a VHV wants to evaluate whether the campaign was effective. Which indicator most directly measures the outcome of the campaign? | A) Number of VHVs who attended the planning meeting  B) Amount of budget spent on larvicide  C) Number of households that eliminated stagnant water containers following the campaign  D) Number of posters distributed in the community |
| **✅ Answer: C** *Rationale: Outcome evaluation requires measuring actual behaviour change resulting from the intervention. The number of households eliminating breeding sites directly reflects the campaign's impact on disease prevention practice.* | |
| **Item 13** A VHV completes a disease surveillance report using the Smart VHV application but realises the data entered contains an error. What is the most appropriate action? | A) Submit the report with the error and correct it verbally when speaking to the health officer  B) Delete the application and reinstall it  C) Use the application's correction or edit function to amend the data before final submission, or contact the subdistrict health-promoting hospital to request correction  D) Submit a separate paper report with the correct information and disregard the digital report |
| **✅ Answer: C** *Rationale: Data accuracy is essential for disease surveillance. The correct procedure is to use the available correction function within the system or to formally notify health authorities of the error to ensure the surveillance database reflects accurate information.* | |
| **Item 14** A VHV is conducting the annual community health needs assessment. Which source of information is most appropriate for identifying the community's current communicable disease priorities? | A) Personal opinion of the VHV based on past experience only  B) Disease surveillance data from the subdistrict health-promoting hospital combined with community input gathered through household visits  C) National disease statistics from the Ministry of Public Health website without local verification  D) Information shared by the village headman without consulting community members |
| **✅ Answer: B** *Rationale: A valid community health needs assessment integrates objective local surveillance data with direct community input, ensuring that identified priorities reflect actual community health needs rather than assumptions.* | |
| **Item 15** A VHV has successfully implemented a community health plan to reduce dengue fever incidence. To ensure the plan is sustainable and self-reliant, what is the most important next step? | A) Submit a report to the district hospital and wait for further instructions  B) Document lessons learned, share outcomes with community members, revise the plan based on evaluation findings, and integrate successful activities into the next annual community health plan  C) Discontinue the activities as the immediate goal has been achieved  D) Request external funding to continue the activities indefinitely |
| **✅ Answer: B** *Rationale: Sustainability and community self-reliance require closing the planning cycle: documenting lessons, sharing results with the community, refining the approach, and embedding successful practices into ongoing community health planning — reducing dependence on external support.* | |

**Scoring Guide**

| **Score (items correct)** | **Percentage** | **Level** |
| --- | --- | --- |
| 13–15 | ≥ 80% | High |
| 9–12 | 60–79% | Moderate |
| 0–8 | < 60% | Low |
| **Total score = (number of correct answers ÷ 15) × 100** | | |
| **Note.** This instrument was developed de novo by the research team to assess integrated overall competency in communicable disease prevention and control among Village Health Volunteers (VHVs) in Chaiyaphum Province, Thailand, as part of the SMART VHV Plus Model evaluation (Phase 3). Items assess competency across three domains (knowledge, skills, and practices) and five SMART components (S, M, A, R, T). Content validity was assessed by three subject-matter experts (IOC range: 0.6–1.0). Copyright belongs to the authors. For use or adaptation, contact the corresponding author. | | |

**Supplementary Table 3.** Key Informant Characteristics and Qualitative Sample Characteristics — Phase 1 Focus Group Discussion Participants (n = 66) and In-Depth Interview Key Informants (n = 34)

| **Key Informant Type** | | | | **Level** | | **n** |
| --- | --- | --- | --- | --- | --- | --- |
| **Focus Group Discussion** | | | | | | |
| **District** | | **FGD Group** | | **Participant Type** | | **n** |
| Khon San | | Group 1 | | VHVs | | 22 |
| Kaeng Khro | | Group 2 | | VHVs | | 22 |
| Chaturat | | Group 3 | | VHVs | | 22 |
| **Total** | | **3 groups** | | | | **66** |
| **Note.** Focus group discussions were conducted separately in three purposively selected districts representing diverse geographic and epidemiological contexts within Chaiyaphum Province. Each group comprised 22 Village Health Volunteers selected through purposive sampling based on active VHV registration status and willingness to participate. Sessions were conducted in Thai at community venues, lasted approximately 60–90 minutes, were audio-recorded with participants' written consent, and were subsequently transcribed verbatim. Topics addressed: communicable disease risk factors, existing surveillance and prevention systems, and prior VHV competency development experiences. | | | | | | |
| **In-depth interviews** | | | | | | |
| **Participant Type** | | | | **Level** | | **n** |
| Public health officers (communicable disease control unit) | | | | Provincial | | 2 |
| Public health officers (primary health care unit) | | | | Provincial | | 2 |
| District public health officers | | | | District | | 4 |
| Subdistrict health-promoting hospital directors | | | | Subdistrict | | 6 |
| VHV presidents | | | | Provincial | | 1 |
| VHV presidents | | | | District | | 3 |
| VHV excellence awardees | | | | Community | | 6 |
| Subdistrict administrative officers | | | | Subdistrict | | 4 |
| Disease surveillance officers | | | | Provincial | | 2 |
| Disease surveillance officers | | | | District | | 2 |
| Disease surveillance officers | | | | Subdistrict | | 2 |
| **Total** | | | |  | | **34** |
| **Note.** Key informants were purposively selected to ensure representation across all levels of the health system and community governance structures relevant to VHV operations in Chaiyaphum Province, Thailand. All 34 key informants who participated in Phase 1 in-depth interviews subsequently participated in Phase 2 participatory model development workshops (retention rate = 100%). | | | | | | |
| **Theme** | **Subtheme** | | **Example Quote** | | **Interpretation** | |
| 1. Knowledge and Situational Assessment | Limited understanding of epidemiology | | "I know how to prevent dengue, but I'm not sure how to assess outbreak situations." (VHV No. 3) | | VHVs lacked theoretical understanding of epidemiological principles and situational analysis. | |
|  | Difficulty interpreting data | | "Numbers and statistics are hard for me to explain." (VHV No. 6) | | Low confidence in using quantitative information for risk assessment. | |
| 2. Data Recording and Management | Manual record-keeping | | "I usually write everything in my notebook." (VHV No. 1) | | VHVs relied on non-standard methods, leading to inconsistent and hard-to-track data. | |
|  | Lack of data system use | | "We don't have a proper system to record cases." (VHV No. 6) | | Absence of systematic data management limited planning and reporting accuracy. | |
| 3. Digital Literacy and Technology Use | Difficulty using smartphones and apps | | "I asked my daughter to send the report online." (VHV No. 3) | | Older VHVs depended on family members due to limited digital skills. | |
|  | Poor access to internet/data | | "I couldn't send reports because my internet ran out." (VHV No. 5) | | Connectivity issues created barriers to timely digital reporting. | |
| 4. Risk Communication and Public Education | Limited communication confidence | | "I'm afraid I might give wrong information." (VHV No. 2) | | VHVs lacked confidence in communicating disease risks effectively. | |
|  | Difficulty addressing misinformation | | "People sometimes believe rumors more than us." (VHV No. 7) | | Inadequate skills in countering misinformation and delivering clear messages. | |
| 5. Leadership and Teamwork | Following directives rather than leading | | "We just wait for instructions from the health centre." (VHV No. 8) | | VHVs showed limited initiative and strategic thinking in community planning. | |
|  | Weak motivational role | | "It's hard to get villagers to join activities." (VHV No. 3) | | Limited leadership hindered community engagement and collaboration. | |
| **Note.** Quotations represent VHV perspectives derived from focus group discussions only. Key informant perspectives from in-depth interviews (n = 34) provided corroborating evidence and are integrated into the narrative analysis in Section 4.1. | | | | | | |

| **Supplementary Table 4.** SMART VHV Plus Model — Detailed Training Programme Documentation. | |
| --- | --- |
| **Programme 1: Advanced Disease Prevention & Control Training**  **(SMART Component: S — Screen)** | |
| Model Component | Communicable disease control |
| SMART Letter | S = Screen |
| Operational Domain | Communicable disease surveillance and at-risk group screening |
| Training Objectives | (1) Apply epidemiological concepts to community-level communicable disease surveillance; (2) Conduct systematic case-finding and at-risk group screening using standardised VHV protocols; (3) Implement the 5-step VHV outbreak response protocol (preparation in the area, case finding and screening, at-risk group registry management, symptom monitoring and follow-up, reporting of results) |
| Session Content | Topic 1: Epidemiological principles and disease transmission dynamics (dengue fever, COVID-19, hand-foot-and-mouth disease, diarrhoeal diseases); Topic 2: Community-level disease surveillance systems and VHV roles within the national Surveillance and Rapid Response Team (SRRT) structure; Topic 3: Case-finding techniques and at-risk group identification criteria; Topic 4: At-risk group registry management and documentation procedures; Topic 5: The 5-step VHV outbreak response workflow — preparation, screening, registry, follow-up, reporting |
| Teaching Methods | Morning session (approximately 3 hours): Lecture and facilitated discussion covering epidemiological concepts and surveillance systems. Afternoon session (approximately 3–4 hours): Scenario-based exercises using simulated outbreak case studies; field simulation exercise practising case-finding and at-risk group screening in the community setting |
| Session Duration | 1 day (approximately 6–8 hours) |
| Trainers | 1 university faculty member in public health (communicable disease epidemiology) + 1–2 district public health officers with expertise in communicable disease surveillance and SRRT operations |
| Training Materials | Presentation slides; integrated participant handbook (Section 1); case study worksheets; at-risk group registry form templates |
| Fidelity Assessment | A domain-specific pre-test was administered immediately before session commencement. The integrated overall competency pre-test (15 items; range: 0–100%) was additionally administered at this point as the overall pre-intervention baseline for the full programme evaluation (Table 3, main manuscript). A parallel domain-specific post-test was administered immediately following session completion. Structured observation checklist completed by research team member during session; post-session participant feedback form assessing relevance, clarity, and self-perceived learning outcomes. |
| PAOR Modifications | Content on dengue surveillance simplified following participant feedback that SRRT-level protocols were perceived as too advanced; additional practice time allocated for registry form completion based on observation of participant difficulty |
| **Programme 2: Management Training Programme**  **(SMART Component: M — Management)** | |
| Model Component | Management |
| SMART Letter | M = Management |
| Operational Domain | Participatory resource management and collaborative problem-solving |
| Training Objectives | (1) Apply participatory management principles to community health activity planning; (2) Plan and allocate health resources and budgets for community disease prevention activities; (3) Facilitate collaborative problem-solving processes within VHV teams and with community stakeholders |
| Session Content | Topic 1: Principles of participatory management and community engagement in health; Topic 2: Resource identification, planning, and budget allocation for community health activities (sources: local administrative organisations, National Health Security Office fund); Topic 3: Collaborative problem-solving frameworks applicable to communicable disease prevention; Topic 4: Documentation and reporting of management activities; Topic 5: Case studies of participatory management in Thai community health contexts |
| Teaching Methods | Morning session (approximately 3 hours): Lecture covering participatory management theory and resource planning frameworks; group discussion of local management challenges identified in Phase 1. Afternoon session (approximately 3–4 hours): Case-based learning using real community health scenarios; role play exercises practising facilitation of collaborative problem-solving meetings |
| Session Duration | 1 day (approximately 6–8 hours) |
| Trainers | 1 university faculty member in public health (community health management) + 1–2 district public health officers with expertise in community health programme management and budget administration |
| Training Materials | Presentation slides; integrated participant handbook (Section 2); resource planning worksheets; budget template forms; case study materials |
| Fidelity Assessment | A domain-specific pre-test was administered immediately before session commencement; a parallel post-test was administered immediately following session completion to assess immediate learning gains. Structured observation checklist completed by research team member during session; post-session participant feedback form assessing relevance, clarity, and self-perceived learning outcomes. |
| PAOR Modifications | Role play scenarios revised to reflect local administrative structures and budget sources specific to Nong Bua Ban Village following reflection session feedback that generic scenarios were less relatable |
| **Programme 3: VHV 4.0 Plus Technology Training**  **(SMART Component: A — Application)** | |
| Model Component | Technology |
| SMART Letter | A = Application |
| Operational Domain | Digital technology application and health information systems |
| Training Objectives | (1) Use smartphones independently for disease reporting via the Smart VHV application and online reporting systems; (2) Access and navigate digital health platforms and internet-based reporting systems in accordance with Ministry of Public Health digital literacy guidelines (VHV 4.0 initiative); (3) Critically evaluate online health information and identify misinformation |
| Session Content | Topic 1: Overview of Thailand's VHV 4.0 digital health initiative and national digital literacy policy for VHVs; Topic 2: Smartphone operation fundamentals (for participants with limited prior experience); Topic 3: Smart VHV application — installation, navigation, and disease report submission; Topic 4: Online reporting systems — data entry, submission, and error correction procedures; Topic 5: Health information literacy — evaluating credibility of online health information and identifying misinformation |
| Teaching Methods | Full-day hands-on workshop format. Morning session (approximately 3 hours): Trainer demonstration of Smart VHV application and online reporting systems using projected display; guided step-by-step practice on individual smartphones. Afternoon session (approximately 3–4 hours): Peer-assisted learning pairs (more digitally experienced VHVs supporting less experienced peers); independent practice exercises completing simulated disease reports; group discussion of health information literacy scenarios |
| Session Duration | 1 day (approximately 6–8 hours) |
| Trainers | 1 university faculty member in public health (digital health) + 1–2 district public health officers with expertise in digital health reporting systems and VHV 4.0 implementation |
| Training Materials | Presentation slides; integrated participant handbook (Section 3); step-by-step smartphone guide (large font format for older participants); Smart VHV application demonstration account; practice worksheets for simulated report completion |
| Fidelity Assessment | A domain-specific pre-test was administered immediately before session commencement; a parallel post-test was administered immediately following session completion to assess immediate learning gains. Practical skills checklist additionally used to assess whether each participant could independently complete a simulated disease report by end of session. Structured observation checklist; post-session participant feedback form. |
| PAOR Modifications | Step-by-step smartphone guide reformatted with larger font and simplified language following observation that participants aged ≥ 50 years experienced difficulty following the original guide; peer-assisted learning pairs introduced after reflection session identified that peer support was more effective than trainer-only instruction for this age group |
| **Programme 4: Leadership & Teamwork Development Programme**  **(SMART Component: R — Response)** | |
| Model Component | Leadership and teamwork |
| SMART Letter | R = Response |
| Operational Domain | Community-level outbreak response and risk communication |
| Training Objectives | (1) Demonstrate leadership in community health contexts using the Four-Directions leadership theory (a leadership framework identifying four directional styles — directing, coaching, supporting, and delegating — applied contextually based on follower readiness and task complexity) applied to VHV roles; (2) Apply teamwork and change management strategies to coordinate community health activities; (3) Motivate and sustain community participation in disease prevention and health promotion campaigns |
| Session Content | Topic 1: Leadership concepts and leadership styles relevant to community health work; Topic 2: Four-Directions leadership theory (a framework comprising four leadership styles: directing, coaching, supporting, and delegating, each applied according to the readiness level of community members and the complexity of the health task at hand) applied to VHV community mobilisation contexts; Topic 3: Change management principles for introducing new health practices in communities; Topic 4: Team coordination strategies for VHV networks within subdistrict health systems; Topic 5: Risk communication principles — communicating health risks clearly, managing misinformation, and sustaining community trust |
| Teaching Methods | Morning session (approximately 3 hours): Lecture on leadership theory and risk communication principles; facilitated group discussion applying Four-Directions theory to participants' own community health roles. Afternoon session (approximately 3–4 hours): Experiential learning activities — group leadership challenges simulating community mobilisation scenarios; self-reflection exercises identifying personal leadership strengths and development areas; peer feedback activity |
| Session Duration | 1 day (approximately 6–8 hours) |
| Trainers | 1 university faculty member in public health (community health leadership) + 1–2 district public health officers with expertise in community mobilisation and health communication |
| Training Materials | Presentation slides; integrated participant handbook (Section 4); Four-Directions leadership self-assessment tool; scenario cards for experiential learning activities; self-reflection worksheets |
| Fidelity Assessment | A domain-specific pre-test was administered immediately before session commencement; a parallel post-test was administered immediately following session completion to assess immediate learning gains. Leadership confidence self-assessment additionally completed by each participant at end of session. Structured observation checklist; post-session participant feedback form. |
| PAOR Modifications | Theoretical content on leadership theory reduced and scenario-based experiential activities expanded following reflection session feedback that participants found abstract theory less engaging; risk communication content repositioned as a standalone topic after observation identified it as the most practically relevant component for participants |
| **Programme 5: Community Health Plan Development Programme**  **(SMART Component: T — Time)** | |
| Model Component | Community health planning |
| SMART Letter | T = Time (real-time) |
| Operational Domain | Timely, self-reliant community health planning |
| Training Objectives | (1) Conduct a structured community health needs assessment using participatory methods; (2) Develop a self-reliant community health plan (a locally owned, resource-realistic plan enabling communities to address health priorities through their own initiative and resources) incorporating health promotion and communicable disease prevention priorities; (3) Evaluate community health plan implementation outcomes against defined indicators |
| Session Content | Topic 1: Community health needs assessment — methods, tools, and data sources available to VHVs; Topic 2: self-reliant community health planning frameworks — principles of community self-reliance (the capacity of communities to identify their own health needs, mobilise local resources, and implement sustainable health solutions with reduced dependence on external support) in health promotion; Topic 3: Health plan structure — situation analysis, objectives, activities, resources, timeline, and evaluation indicators; Topic 4: Integration of communicable disease prevention priorities into community health plans; Topic 5: Plan evaluation methods — indicator selection, data collection, and reporting to subdistrict health-promoting hospitals |
| Teaching Methods | Full-day participatory workshop. Morning session (approximately 3 hours): Lecture and facilitated discussion on community health needs assessment and self-reliant planning principles; group exercise analysing community health data from Nong Bua Ban Village. Afternoon session (approximately 3–4 hours): Hands-on planning exercise — participants work in small groups to draft a community health plan for their village using a structured template; group presentation of draft plans followed by peer and trainer feedback |
| Session Duration | 1 day (approximately 6–8 hours) |
| Trainers | 1 university faculty member in public health (community health planning) + 1–2 district public health officers with expertise in community health planning and subdistrict health system administration |
| Training Materials | Presentation slides; integrated participant handbook (Section 5); community health plan template; community health data summary (Nong Bua Ban Village); evaluation indicator checklist |
| Fidelity Assessment | A domain-specific pre-test was administered immediately before session commencement; a parallel post-test was administered immediately following session completion to assess immediate learning gains. The integrated overall competency post-test (15 items; range: 0–100%) was additionally administered at this point as the overall post-intervention outcome for the full programme evaluation (Table 3, main manuscript). Plan quality rubric used to assess completeness and feasibility of draft community health plans produced during session. Structured observation checklist; post-session participant feedback form. |
| PAOR Modifications | Community health data provided for the planning exercise updated to reflect current Nong Bua Ban Village context following observation that generic provincial-level data were insufficiently motivating for participants; group presentation component added after reflection session identified that public accountability for plan quality improved participant engagement and plan completeness |

**General Note for All Programmes**

The total intervention spanned 5 training days across April–August 2024, delivered at Nong Bua Ban Subdistrict Health-Promoting Hospital, Chaturat District, Chaiyaphum Province. Each programme was delivered by one university faculty member in public health and one to two district public health officers with domain-relevant professional expertise; no formal training-the-trainer procedure was required as all trainers held professional qualifications in their respective areas. Training materials for all five programmes comprised presentation slides and an integrated participant handbook.

Competency assessment followed a repeated pre-post measurement design. Immediately before each training session, participants completed a domain-specific pre-test; immediately following the session, a parallel post-test was administered to assess immediate learning gains. This procedure was repeated for each of the five programmes independently. The overall pre- and post-intervention competency scores reported in Table 3 of the main manuscript represent the integrated competency performance test (15 items; range: 0–100%) administered before Programme 1 (overall pre-intervention baseline) and after Programme 5 (overall post-intervention outcome) respectively.

Programme fidelity was monitored through structured observation checklists completed during each session and post-session participant feedback forms. Modifications made between PAOR cycles are documented in the PAOR Modifications row for each programme above and described in Section 4.2 of the main manuscript. The SMART VHV Plus Model is an original framework developed within this study; training materials are available from the corresponding author on reasonable request.
